# Catch-Up Saccades in Vestibular Hypofunction: A Contribution of the Cerebellum?

**DOI:** 10.1007/s12311-023-01512-w

**Published:** 2023-01-21

**Authors:** Ruben Hermann, Camille Robert, Vincent Lagadec, Mathieu Dupre, Denis Pelisson, Caroline Froment Tilikete

**Affiliations:** 1 Université Claude Bernard Lyon 1, CNRS, INSERM, Centre de Recherche en Neurosciences de Lyon CRNL U1028 UMR5292, IMPACT, F-69500 Bron, France; 2 Lyon I University, Lyon, France; 3 Cervico-Facial Surgery and Audiophonology, Hospices Civils de Lyon, ENT, Hôpital Edouard Herriot, Lyon, France; 4 French Vestibular Rehabilitation Society, Lyon, France; 5 Neuro-Ophthalmology Unit, Hospices Civils de Lyon, Hopital Neurologique Et Neurochirurgical P Wertheimer, Lyon, France

**Keywords:** Vestibular areflexia, Bilateral vestibulopathy, Head-impulse test, Catch-up saccades, Cerebellar dysfunction, CANVAS

## Abstract

Long-term deficits of the vestibulo-ocular reflex (VOR) elicited by head rotation can be partially compensated by catch-up saccades (CuS). These saccades are initially visually guided, but their latency can greatly decrease resulting in short latency CuS (SL-CuS). It is still unclear what triggers these CuS and what are the underlying neural circuits. In this study, we aimed at evaluating the impact of cerebellar pathology on CuS by comparing their characteristics between two groups of patients with bilateral vestibular hypofunction, with or without additional cerebellar dysfunction. We recruited 12 patients with both bilateral vestibular hypofunction and cerebellar dysfunction (BVH-CD group) and 12 patients with isolated bilateral vestibular hypofunction (BVH group). Both groups were matched for age and residual VOR gain. Subjects underwent video head impulse test recording of the horizontal semicircular canals responses as well as recording of visually guided saccades in the step, gap, and overlap paradigms. Latency and gain of the different saccades were calculated. The mean age for BVH-CD and BVH was, respectively, 67.8 and 67.2 years, and the mean residual VOR gain was, respectively, 0.24 and 0.26. The mean latency of the first catch-up saccade was significantly longer for the BVH-CD group than that for the BVH group (204 ms vs 145 ms, *p* < 0.05). There was no significant difference in the latency of visually guided saccades between the two groups, for none of the three paradigms. The gain of covert saccades tended to be lower in the BVH-CD group than in BVH group (*t* test; *p* = 0.06). The mean gain of the 12° or 20° visually guided saccades were not different in both groups. Our results suggest that the cerebellum plays a role in the generation of compensatory SL-CuS observed in BVH patients.

## Introduction

During high velocity head rotations, gaze stabilization is mainly achieved through the vestibulo-ocular reflex (VOR). In case of vestibular loss, this slow eye movement reflex can be compensated by catch-up saccades (CuS) that redirect gaze toward the target [1]. In the early stages of a vestibular deficit, patients mostly produce visually guided catch-up saccades to bring the image of the target back onto their fovea after the head movement (hence also called overt saccades). When vestibular function does not recover, some of these catch up saccades show a reduced latency to the extent that they unfold during the head movement, hence also called covert saccade or short-latency catch up saccades (SL-CuS) [2]. Both the short latency and the regularity of these SL-CuS have been shown to be linked to better dynamic visual acuity and to have positive functional impact [3, 4].

These SL-CuS can occur as early as 70 ms after the beginning of the head movement[5]. As the shortest latency of visually guided saccades is around 100 ms (express saccades) [6], this raises the questions regarding what actually triggers these SL-CuS. Different sources of information participating in the triggering of SL-CuS could include the proprioceptive cervical receptors, residual vestibular function (in particular from the contralateral ear), cognitive function, visual information, and multisensory integration [5, 7–10]. Beside the triggering signals, the neural circuits which underlie these quick compensatory catch-up saccades are also still unknown.

The cerebellum is crucially involved in general motor control, including eye movements control, and particularly in plastic modifications of oculomotor responses such as adaptation, learning, and compensation [11]. Save for some exceptions [12, 13], the cerebellum does not play a major role when it comes to saccade latency. It is nevertheless crucial for vestibular and saccadic adaptation [14]. Additionally, it is strongly connected to the vestibular nuclei. Therefore, beyond the demonstrated contribution of the cerebellum to theVOR adaptation mechanisms in case of chronic vestibular deficit, this structure could also participate in this critical gaze stabilization function by contributing to the generation of SL-CuS.

One way to demonstrate the role of the cerebellum in this compensatory mechanism is to study gaze stabilization in patients presenting with both vestibular and cerebellar deficits, like patients with cerebellar ataxia neuropathy vestibular areflexia syndrome (CANVAS). Our hypothesis is that cerebellar dysfunction in these patients could hinder the shortening of CuS latency as compared to patients with vestibular deficits only.

The main objective of this study was to evaluate this hypothesis by measuring the impact of cerebellar dysfunction on CuS in patients with cerebellar ataxia and vestibular hypofunction. In the first experiment, we recorded VOR and CuS during head impulse test in two groups of patients with bilateral vestibular hypofunction of which one had additional cerebellar dysfunction.

In a second experiment, performed in two similar groups of patients, we aimed to better understand the specificity of cerebellar contribution to CuS by comparing the latency of purely visually guided saccades.

## Material and Methods

This prospective study was performed in the neuro-ophthalmology unit of the Lyon University Hospital between November 2019 and March 2022.

We recruited patients with bilateral vestibular hypofunction and cerebellar dysfunction (BVH-CD group) and patients with isolated bilateral vestibular hypofunction (BVH group). Data of 6 previously recorded patients (2016) were used for the BVH group [3].

All subjects met the clinical BARANY criteria A, B, C, and D. For criteria C, we only used vHIT as our study focused on compensation during high velocity head movements.

In order to reduce biases while comparing the latency of the first catch up saccade between the group of BVH patients and the group of BVH-CD patients, we aimed for these two groups to be as comparable as possible. As age has been shown to affect SL-CuS in patients with bilateral vestibular hypofunction [3], we matched mean age across our BVH-CD group and our BVH group. Even though it has not been shown that residual vestibulo-ocular reflex (VOR) impacts CuS latencies, we also chose to match residual vestibular function across both groups, based on the mean VOR gain measured during vHIT of the lateral semicircular canals.

### BVH-CD Group

Inclusion criteria were:

- Age between 18 and 90
- Bilateral vestibular hypofunction (BVH) as defined by the Barany Society criteria [15]

Cerebellar dysfunction:

- Obvious cerebellar atrophy on MRI
- At least one of the following clinical manifestations: postural and segmental cerebellar dysmetria, cerebellar dysarthria, specific cerebellar oculomotor disorders (at least one of the following: saccade hypermetria, downbeat nystagmus, obvious deficit of the visual inhibition of the visuo-vestibulo-ocular reflex)

Subjects were not included if they had other underlying central neurological condition, otological disorder other than vestibular hypofunction, corrected standard visual acuity lower than 5/10 Snellen equivalent, ocular motor palsy, ocular instability in primary gaze position, instability of the cervical spine, or if they were taking drugs interfering with eye movements.

A total of 12 subjects were included in the BVH-CD group. Ten patients had cerebellar ataxia neuronopathy and vestibular areflexia syndrome (CANVAS) with positive RFC1 testing. Two patients had chronic progressive cerebellar dysfunction associated with bilateral vestibular hypofunction, of unknown origin. Clinical manifestation of cerebellar oculomotor disorders as well as other clinical cerebellar manifestation are shown in Table 1.

**Table 1:** Clinical features of cerebellar manifestations of the 12 subjects of the BVH-CD group: J_*SP*, jerky smooth pursuit; *J_VVOR*, jerky visuo-vestibular reflex; *GEN*, gaze evoked nystagmus; *DBN*, down beat nystagmus; *HS*, hypermetric saccades; *SWJ*, square wave jerks; L_*DyM*, limb dysmetria; *WG*, wide gait; *DyA*, dysarthria; *Ad*, adiadochokinesia

Subject	J_SP	J_VVOR	GEN	DBN	HS	SWJ	L_DyM	WG	DyA	Ad
1	✓	✓	✓	✓			✓			
2	✓	✓	✓	✓			✓	✓		✓
3	✓	✓	✓				✓	✓	✓	
4	✓	✓					✓	✓	✓	✓
5	✓	✓	✓		✓		✓	✓		
6	✓	✓	✓	✓	✓		✓	✓	✓	
7	✓	✓					✓	✓		
8	✓	✓				✓				
9	✓	✓	✓	✓			✓	✓	✓	✓
10	✓	✓	✓				✓			
11	✓	✓	✓	✓	✓		✓	✓	✓	
12	✓	✓	✓	✓		✓		✓		✓

### BVH Group

Inclusion criteria were:

- Age between 18 and 90
- Bilateral vestibular hypofunction (BVH) as defined by the Barany Society criteria [15]

Exclusion criteria were similar to the other group with the exception of the exclusion in case of any underlying neurologic disorder.

A total of 12 subjects were included in the BVH group. Etiologies were the following: 5 idiopathic, 4 iatrogenic (aminoglycoside); 1 Meniere’s disease, 1 post infectious, 1 bilateral vestibular neuritis.

### Material

#### Video Head Impulse Test (vHIT)

Head and eye movements during head impulse test were recorded using a lightweight portable vHIT device (Hardware: ICS Impulse, GN Otometrics, Taastrup, Denmark; Software: Otosuite Vestibular software). Head movements were recorded with a nine-axis motion sensor. Movements of the right eye were recorded with a high-velocity infrared camera. Both the head motion-sensor and eye camera were mounted on a lightweight frame and run at a 250 Hz sampling rate. The infrared camera was calibrated by having the subject gaze toward two light dots projected at eye level on the wall from built-in lasers.

#### Eye tracker

Visually guided saccades were recorded using high-velocity infrared cameras (EyeBRAIN® tracker now distributed by SURICOG Company, Paris, France). Movements of both eyes were recorded with a sampling rate of 300 Hz and an accuracy of 0.5°. Eye-tracker was calibrated with a build in 9-point calibration system.

### Method

#### Head Impulse Test (vHIT)

This evaluation procedure has been described in details in a previous study[3]. In short, a single experienced examiner standing behind the subject performed outward horizontal impulses. Subjects had to gaze toward a target located 2 m in front of them. Their head was tilted forward to align the plane of their horizontal semicircular canals with the horizontal plane. A minimum of 10 valid horizontal head impulses (head speed > 200°/s) was achieved in each direction. Head and eye velocity data were then exported in CSS format for off line analysis.

#### Visually Guided Saccades (VGS)

Horizontal visually guided saccades were recorded in 3 different conditions: STEP, GAP, and OVERLAP. In all conditions, participants were seated in a dimly lit room facing a computer screen 60 cm in front of them. Their head was stabilized using a head and chin rest. For each trial, subjects first had to look at a dot in the center of the screen. Then they fixated a second dot as soon as it appeared at 12° or 20° from the central dot, either to the left or the right. After 1000 ms, the second dot disappeared, and the central dot appeared again for 2400 to 3600 ms until the next trial started.

For the STEP condition, the central dot disappeared at the same time as the second (lateral) dot appeared. For the GAP condition, the central dot disappeared 200 ms before the lateral dot appeared. For the OVERLAP condition, the central dot never disappeared. In each paradigm, twelve randomized outward saccades (6 to the left and 6 to the right) were recorded at 12° and at 20°. Thus, a total of 72 visually guided saccades were recorded.

#### Analysis of Head and Eye Movements

Data analysis for both vHIT and VGS was done in a program developed in our lab and running on MATLAB v.8.1 (MathWorks, MA, USA).

Details of the vHIT analysis have already been published [3]. In short, movements were first identified automatically and differentiated from artefacts by using a 5°/s head or eye velocity threshold. Then for each identified movement, five cursors defined, respectively, the starting and ending positions, the starting and ending times, and the time of maximum velocity. Each automatically detected movement was checked and cursors could be manually adjusted. In line with the previously described protocol, we chose to identify a maximum of three eye movements per head impulse (vestibulo-ocular reflex and up to 2 catch-up saccades). Covert saccades (CS) were defined as saccades occurring before the end of the head movement and overt saccades (OS) after the end of the head movement. The first catch-up saccades were defined as the first corrective saccade and could correspond to covert or overt saccades. For each head impulse, VOR gain was calculated as the amplitude ratio between eye movement and head movement at the end of VOR sequence. The end of the VOR sequence was defined as either the start of the covert saccade or the maximum point of VOR amplitude in case of absence of covert saccades. Saccadic gain was calculated as the ratio of eye movement amplitude during CS or OS and of total head movement amplitude. Latencies were calculated as the onset time of each eye movement (VOR, CS, OS) relative to the beginning of head movement. For each subject, the occurrence of CS (frequency in percent) was determined as the total number of CS relative to the total number of head impulses. The consistency of CS initiation was determined by the mean of individual standard deviation of the latency.

For visually guided saccades, analysis was done on both eyes. Eye movements were identified automatically by using an eye velocity threshold of 30°/s to differentiate movements from artefacts. Cursors were manually adjusted if necessary. We analyzed the latency, amplitude, and gain of primary saccades and only the amplitude and gain of secondary saccades by using the average between the values of both eyes. Gain was defined as the ratio between the actual saccade amplitude and the amplitude required to capture the target. Latency was defined as the difference in time between the appearance of the second dot and the beginning of the eye movement. Primary saccades measured in STEP condition were considered accurate if their amplitude was 12° + / − 1 for a target amplitude of twelve degrees or 20° + / − 2 for a target amplitude of twenty degrees; they were considered hypometric or hypermetric if their amplitude was below or above these thresholds, respectively.

### Statistical Analysis

All data were stored and analyzed using JASP (JASP Team, Version 0.16.2, 2022). Statistical analyses were done using independent samples Student *t* test, Mann–Whitney test, or ANOVA depending on the normality of the distribution, as established by Shapiro–Wilk test, and the number of variables tested. All tests were two-tailed, and a statistical threshold *p* value < 0.05 was used.

## Results

### Experiment 1: Video Head Impulse Test

Twelve subjects were included in each group with respectively 9 and 8 males for the bilateral vestibular hypofunction and cerebellar dysfunction group (BVH-CD) and the bilateral vestibular hypofunction group (BVH). The mean age in the BVH-CD and BVH groups was, respectively, 67.8 years (SD 5.6) and 67.2 years (SD 6.9) (*t* test; *p* = 0.80). The mean residual VOR gain in the BVH-CD and BVH groups was, respectively, 0.24 (SD 0.15) and 0.26 (SD 0.15) (*t* test *p* = 0.78).

**Fig. 1:**
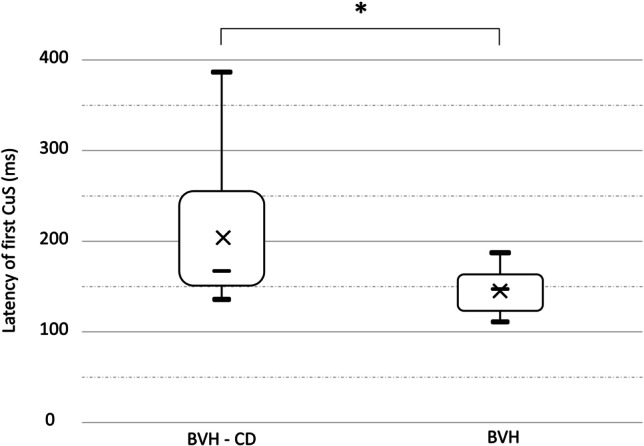
Boxplots of the latency of the first catch-up saccade (CuS) during vHIT showing lower

*The mean latency of the first catch-up saccade* differed significantly between the BVH-CD group (204 ms, SD 75 ms) and the BVH group (145 ms, SD 26 ms) (Mann–Whitney; *p* = 0.04). Details on latency of the first catch-up saccade (CuS) are shown in Fig. 1.

extreme, 1st quartile, mean (cross), median (horizontal line), 3rd quartile, and upper extreme. *: statistically significant difference between the two groups (*p* < 0.05).

Furthermore, the standard deviation of the latency of the first CuS was significantly greater in the BVH-CD group (49.6 ms) than in the BVH group (31.2 ms) (Mann-Withney; *p* = 0.043).

The mean latency of covert saccades did not differ between the BVH-CD group (132 ms) and the BVH group (124 ms) (*t* test; *p* = 0.43). Note that the gain of covert saccades tended to be lower in the BVH-CD group (0.33; SD 0.1) than in BVH group (0.45, SD 0.14) (*t* test; *p* = 0.06). The covert saccades did not differ in mean frequency between the two groups (*t* test; *p* = 0.39), being present in 52% of trials in BVH-CD patients and 63% of trials in BVH patients.

Finally, the mean latency of *overt saccades* was also similar in the BVH-CD group (264 ms) and the BVH group (257 ms) (*t* test; *p* = 0.73).

### Experiment 2: Visually Guided Saccades

#### Primary Saccade Latency

Ten subjects underwent testing of visually guided saccades from each group. The mean age was, respectively, 69.8 (SD 9) and 70.0 (SD 6) for these BVH and BVH-CD sub-groups (*t*(18) = 0,000; *p* = 1,000). Eight men and 2 women participated in each group.

A three-way ANOVA (factors group x paradigm x eccentricity) did not reveal any significant effect of group on the latency of the primary visually guided saccades ((*F*(1,18) = 0.006; *p* = 0.941) and no significant interaction between group and paradigm ((*F*(2,36) = 0.720; 0.494) and between group, paradigm, and eccentricity ((*F*(2,36) = 0.246; 0.783). As expected, there was a significant effect of both eccentricity (*F*(1,18) = 8.029; *p* = 0.011) and paradigm (*F*(1,18) = 67.841; *p* < 0.001) on the latency of the first primary visually guided saccade.

**Fig. 2:**
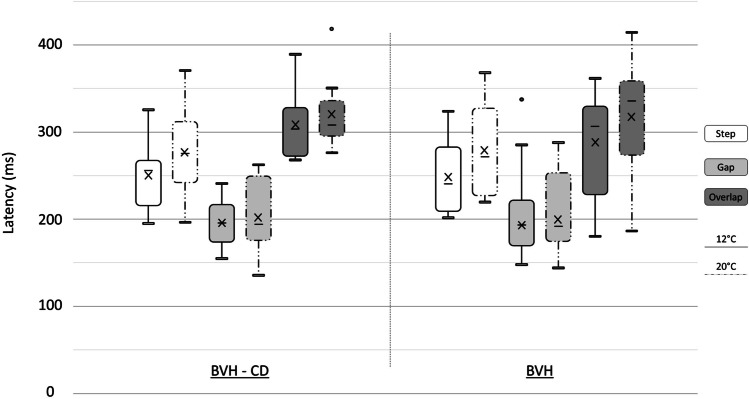
Boxplots of the latency of primary visually guided saccades in the step, gap, and overlap conditions at 12° and 20° for BVH and BVH-CD patients, showing lower extreme, 1st quartile, mean (cross), median (horizontal line), 3rd quartile, and upper extreme. The two dots indicate extreme outliers

Detailed results of the latency data are shown in Fig. 2.

The mean gain of the visually guided saccade to the 12° target was, respectively, 0.88 (SD 0.14) and 0.96 (SD 0.05) for BVH-CD and BVH (*t* test *p* = 0.20). Saccades to the 20° target reached a mean gain of, respectively, 0.92 (SD 0.10) and 0.96 (SD 0.03) for BVH-CD and BVH (Mann–Whitney *p* = 0.43). For the 12° target, hypometric saccades were more frequent in the BVH-CD group than in the BVH group with, respectively, 47% and 20% (Mann–Whitney *p* = 0.04). Conversely for the 20° target, hypermetric saccades were more frequent for the BVH-CD group than for the BVH group with, respectively, 8% and 0% (Mann–Whitney *p* = 0.04).

## Discussion

The main goal of this study was to evaluate the impact of cerebellar dysfunction on the latency of compensatory catch-up saccades (CuS) during head impulse testing in patients with bilateral vestibular hypofunction (BVH). We found that the first catch up saccade had a significantly longer latency in patients with BVH and cerebellar dysfunction (BVH-CD) than in patients with only BVH. Additionally, the triggering of these first saccades was disorganized in BVH-CD patients as shown by an increased standard deviation of the latency when compared to BVH patients. In contrast, the latency of visually guided saccades in STEP, GAP, and OVERLAP conditions was similar between both groups of patients. Together, these results suggest that the cerebellum contributes to the compensatory response which is observed in BVH patients and which consists of a reduced latency of catch-up saccades and thus of the emergence of short-latency CuS (SL-CuS).

Mean residual gain of the lateral canals VOR in the BVH-CD population was 0.24 (SD 0.15) and in the BVH population 0.26 (SD 0.15). These values are close to the 0.28 mean VOR gain we had published in a previous study on 20 patients with BVH[16].

When looking at all covert saccades together, i.e., compensatory saccades occurring before the end of head movement, no significant difference of latency has been observed between the BVH and BVH-CD patient’s groups. This is to be expected as, according to their definition according to the head movement, covert saccades do not represent a neurophysiological entity but rather constitute a heterogeneous category of saccades. For example, a saccade occurring at 180 ms could potentially be called covert saccade if the duration of the head movement is 185 ms or overt saccade if the duration of the head movement is 175 ms. For this reason, we emphasized on analyses of the first compensatory catch-up saccade (CuS), which could be covert or overt. The threshold for discriminating between the different types of CuS (short or long latency) is yet to be determined but should probably vary with age.

Thus, when focusing on the first CuS, we found that their mean latency (204 ms, SD 75 ms) significantly increased in BVH-CD patients as compared to BVH patients (145 ms (SD 26 ms)). This suggests that the cerebellum plays a role in triggering CuS.

One hypothesis is that dysfunctions associated to cerebellar ataxia in our BVH-CD patient’s population would affect the saccadic network and result in an increased latency of all saccadic responses. Indeed, saccade anomalies are a typical sign of cerebellar lesions, particularly those invading the dorsal cerebellar vermis and posterior fastigial nucleus. These anomalies are mostly related to inaccuracy of saccadic amplitude. Lesions of the dorsal cerebellar vermis impair saccade adaptation, thus disrupting the maintenance or restoration of saccadic accuracy [12, 17–19]. Consistent with this literature, our BVH-CD patients produced a significantly higher percentage of inaccurate (hypometric and/or hypermetric) saccades when compared to the BVH group. Although increased saccadic latency is not a typical sign of cerebellar dysfunction, it has been documented for visually guided saccades after the ablation of the dorsal cerebellar vermis in primates [12] and for anti-saccades in idiopathic cerebellar atrophy [13]. To test the hypothesis that cerebellar dysfunction on its own would be responsible for increased saccadic latency, we analyzed the latency of visually guided saccades in BVH-CD and BVH groups. We did not find any difference of VGS latency between these groups, either for the STEP, GAP, or overlap condition or for the 12° or 20° target eccentricity. The increase in latency of the first CuS during the head impulse test is therefore not due to a general increase in saccade latency linked to an alteration of the saccadic system following cerebellar dysfunction.

Another hypothesis is that BVH associated to cerebellar dysfunction in our patients’ population is due to cerebellar and/or other central dysfunction rather than a peripheral vestibular dysfunction. In that case, BVH in both populations could not be truly compared. Lesions of the cerebellum can profoundly alter slow eye movements in a manner that depends on the lesion location. Typical manifestations of lesions of the flocculus and paraflocculus are impaired smooth pursuit, spontaneous nystagmus, and gaze holding deficit [20, 21] which can also be seen in BVH-CD such as CANVAS[22]. Lesions of the flocculus/paraflocculus region also impair adaptation of the VOR[20, 21, 23, 24] but do not usually lead to loss of vestibular function. In case of CANVAS, vestibular impairment is due to a ganglionopathy of the vestibular nerve [25]. Therefore, the vestibular impairment is probably independent of the cerebellar dysfunction in BVH-CD and more likely corresponds to peripheral vestibular dysfunction comparable to that in BVH patients.

The last hypothesis is that the cerebellum, given its well-known contribution to adaptive changes of motor or sensorimotor functions, drives or helps in the establishment of a SL-CuS within a network involved in gaze stability when vestibular dysfunction remains over the long term. Indeed, the cerebellum plays a fundamental role in motor learning. Following physiological or pathological changes, it participates in the recalibration of various motor or sensorimotor responses and in the preservation of their performance. This cerebellar role has been studied in depth in the case of visuomotor adaptation [26]. In the BVH-CD group, the mean latency of the first CuS during head impulse tests (204 ms) was very similar to the mean latency of VGS in the gap paradigm (206 ms and 212 ms for targets at 12° and 20°, respectively). Based on this similarity, we argue that BVH-CD patients can use the classical visually guided saccades network to generate CuS, but not the saccade network which develops progressively and specifically triggers SL-CuS in BVH patients with preserved cerebellar function. Organization of CuS in vestibular hypofunction probably reflects efficient central compensation and has been shown to be correlated to lower Dizziness Handicap Inventory Scores [27]. We suggest that within the saccade network involved in the establishment of SL-Cus, the cerebellum is probably also responsible for decreasing the variability of the latency of the first CuS as reflected by the difference in standard deviation of this parameter between both groups.

## Conclusion

Patients with added cerebellar dysfunction show an increased latency of catch-up saccades when compared to patients with isolated vestibular deficit, despite the fact that visually guided saccades latency remains similar in both groups. Our results suggest that the cerebellum plays a role in the generation of compensatory short latency catch-up saccades observed during head impulse testing in patients with bilateral vestibular hypofunction. The information of the role of the cerebellum in the neural network of compensatory catch-up saccade is one important step in understanding the occurrence of short-latency catch up saccades. Other studies analyzing the role of proprioceptive cervical receptors, residual vestibular function, cognitive function, visual information, and multisensory integration will help to desiccate this network.

## Data Availability

The datasets used in this study can be accessed if needed by contacting the corresponding author.
