# Correction to: Catch-Up Saccades in Vestibular Hypofunction: A Contribution of the Cerebellum?

**DOI:** 10.1007/s12311-023-01530-8

**Published:** 2023-02-09

**Authors:** Ruben Hermann, Camille Robert, Vincent Lagadec, Mathieu Dupre, Denis Pelisson, Caroline Froment Tilikete

**Affiliations:** 1 Université Claude Bernard Lyon 1, CNRS, INSERM, Centre de Recherche en Neurosciences de Lyon CRNL U1028 UMR5292, IMPACT, F-69500 Bron, France; 2 Lyon I University, Lyon, France; 3 Cervico-Facial Surgery and Audiophonology, Hospices Civils de Lyon, ENT, Hôpital Edouard Herriot, Lyon, France; 4 French Vestibular Rehabilitation Society, Lyon, France; 5 Neuro-Ophthalmology Unit, Hospices Civils de Lyon, Hopital Neurologique Et Neurochirurgical P Wertheimer, Lyon, France

**Correction to: The Cerebellum**

10.1007/s12311-023-01512-w

The original version of this article unfortunately contained mistakes in authorgroup. The authors’ surnames and given names were interchange.

With this, the author list has been corrected from “Hermann Rubenf, Robert Camille, Lagadec Vincent, Dupre Mathieuv Pelisson Denis, Froment Tilikete Caroline” to “Ruben Hermann, Camille Robert, Vincent Lagadec, Mathieu Dupre, Denis Pelisson, Caroline Froment Tilikete”.

The original article has been corrected.

